# The Effectiveness of Online Thematic Expressive Writing on Prolonged Grief Disorder, Anxiety, Depression, and Positive Mental Health among Refugees in the Transition Stage of Asylum Seeking

**DOI:** 10.1007/s10903-025-01778-8

**Published:** 2025-09-12

**Authors:** Nesreen Dababneh, Jürgen Margraf, Fawwaz Ayoub Momani, Lena-Marie Precht, Julia Brailovskaia

**Affiliations:** 1 Mental Health Research and Treatment Center (FBZ), Department of Clinical Psychology and Psychotherapy, Ruhr University, Bochum, Germany; 2 German Center for Mental Health (DZPG), partner site Bochum/Marburg, Bochum, Germany; 3 Department of psychological Counseling, Yarmouk University, Irbid, Jordan

**Keywords:** Expressive writing, Prolonged grief disorder, Positive mental health, Depression, Anxiety

## Abstract

Prolonged grief disorder (PGD) is a recently recognized disorder, and there is a growing need for adapted-related interventions to contexts at higher risk. Bereaved refugees in the transition stages of asylum-seeking due to unavailable mental health services and cultural language barriers necessitate adaptive, accessible PGD interventions. Thematic expressive writing (EW), as a promising guided self-help tool, can increase integrated grief and prevent mental health deterioration. This study aims to investigate the effectiveness of online guided thematic EW based on the dual process model (DPM) of coping with bereavement in decreasing PGD, depression, and anxiety and in increasing positive mental health (PMH). An experimental longitudinal design included 116 adult bereaved refugees. Participants who resided in Jordan were randomly assigned to the experimental groups received one online psychoeducation session, followed by daily online thematic EW tasks using Pennebaker’s writing paradigm over one week. The first group applied tasks related to loss, the second applied restoration-oriented tasks, the third applied a combination of both tasks, and there was one control group. Longitudinal data was collected through online self-report surveys over three time points: baseline, post-intervention, and one-week follow-up. Findings showed that the combination of the loss and restoration-oriented group had a significant within-subject effect on PGD, depression, anxiety, and PMH, while the restoration-oriented group had a significant effect on PGD. However, only PMH improved between the group conditions, and there was no significant interaction effect between time and the experimental conditions. The present results confirm that EW as a scalable intervention for bereaved refugees in the transition stage has a positive but not durable influence on mental health. Guided self-help tools can overcome refugees’ challenges related to accessibility to mental health services and can be a promising, scalable intervention.

**Supplementary Information:**

The online version contains supplementary material available at 10.1007/s10903-025-01778-8.

## Introduction

Prolonged grief disorder (PGD) is a persistent and fluctuating process of grief reactions, characterized by longing and sorrow related to the lost person and the death context (Boelen and Kolchinska 2022; Lenferink et al. 2024). The estimated prevalence is up to 13% in the general population (Comtesse et al. 2024). The context of death related to social, psychological, and biological factors plays an important role in the accommodation of the grief, and it can transform healthy grief into a dysfunctional mental health state (Peña-Vargas et al. 2021). Thus, within certain contexts, such as the refugee a higher risk of developing PGD, has an average of 6 to 54% (Lechner-Meichsner et al. 2024).

Grief within the refugees is also linked to the death related traumatic war experience and to the ongoing displacement-related distress (Lechner-Meichsner et al. 2024; Miller and Rasmussen 2017). Furthermore, different stages of refugee status from the initial flee to final resettlement have different characteristics that shape the mental health status and needs (Miller and Rasmussen 2017; Vukčević Marković et al. 2021). Thus, different stages require adapted psychological interventions that guarantee accessibility. Previous research highlighted the importance of designing specialized scalable interventions addressing distinct patterns of distress caused by refugee experience (Lechner-Meichsner et al. 2024; Nickerson et al. 2014)and the need for innovative interventional research to address PGD across various sociodemographic contexts (Prigerson et al. 2021).

Refugee guideline frameworks for mental health services recommend self-help delivery methods when psychological resources are scarce and inaccessible (Deutsche Gesellschaft für Internationale Zusammenarbeit 2018; Inter-Agency Standing Committee 2007, 2022). Therefore, the current study addresses the efficacy of thematic guided expressive writing (EW) in a self-help delivery form in reducing prolonged grief symptoms and related disorders, and in improving positive mental health (PMH) as an emotional, social and cognitive well-being(Lukat et al. 2016).

EW improves mental health by influencing biopsychosocial and emotional outcomes (Pennebaker 2004). As a self-help tool it contributes to participants’ reduces psychological distress (Asanov et al. 2024), addressing behavior modification within daily real-world contexts (Pennebaker 2004). Self-disclosure through specific writing tasks, such as a letter to the deceased can create a comprehensive narrative, foster a healthy bond with the deceased, and reduce avoidance tendencies (Larsen, 2024). Furthermore, findings on longitudinal writing interventions showed improved coping, reduced anxiety, depression, and prolonged grief in the bereaved group (Den Elzen et al. 2023).

The content of EW can be linked to the dual process model (DPM) of coping with bereavement, by including content focus on the bidirectional coping dynamics that confront primary stressors related to the loss event and secondary stressors associated with life after loss (Stroebe and Schut 1999). Common PGD research focuses on primary stressors related directly to the death event, this model address also the role of secondary stressors as part of a restoration-oriented coping process with life challenges after the loss (Stroebe and Schut 1999, 2010). For refugees, this can include shelter adaptation and assuming new roles and lifestyles in the host communities (Bunn et al. 2023).

The current experimental study investigates the effectiveness of guided, thematic EW designed based on DPM of coping with bereavement in increasing PMH and decreasing prolonged grief, depression, and anxiety among bereaved refugees during transition stages of asylum-seeking.

## Methods

The present study used a longitudinal experimental design over three time points, including three experimental groups and one control group. It was approved by the respective Institutional Review Board (IRB/2024/043).

### Sample

**Fig. 1:**
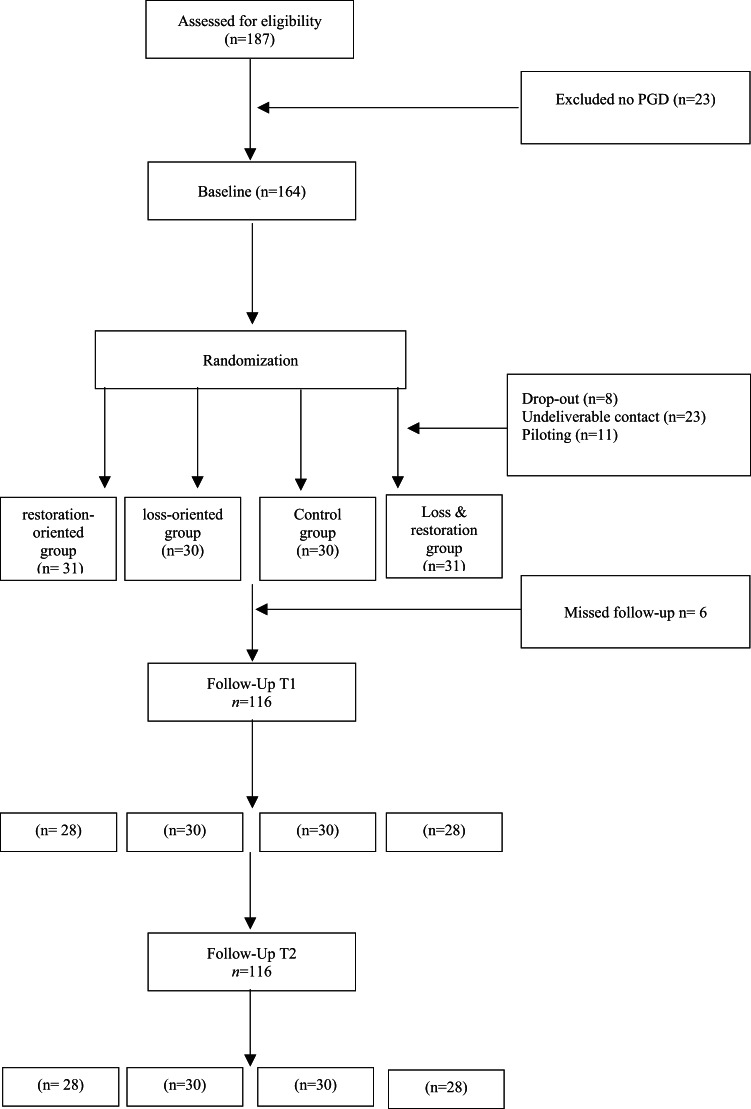
Flow diagram of participant recruitment, missing rate, and attrition within three time points

At baseline, the overall sample consisted of 164 adult bereaved refugees who live in Jordan. Participant recruitment and missing data across three-time points is in the flow diagram in Fig. 1. The screening was conducted for a total of 187 participants, and 164 participants met the inclusion criteria participated in the baseline assessment. The final baseline sample included 122 participants after excluding the dropouts *n* = 8, undeliverable contact information *n* = 23, and a piloting sample *n* = 11. After the randomization assignment to one of four groups based on the Dual Process Model: the loss-oriented group *n* = 30, the restoration-oriented group *n* = 31, the combined loss and restoration-oriented group *n* = 31, and a control group *n* = 30. During the first follow-up, *n* = 6 participants had unreachable contact, resulting in a final sample of *n* = 116. The final sample was distributed across the groups, as shown in Figs. 1 and 28 participants were assigned to the restoration-oriented group, 30 to the loss-oriented group, 28 to the combined group, and 30 to the control group.

Table 1 shows descriptive statistics of socio-demographic characteristics for the overall sample and each group.

**Table 1:** Descriptive statistics of socio-demographic characteristics per group *M* = mean; *SD* = standard deviation

	*N* = 116	Restoration-oriented group *N* = 28	Loss-oriented group *N* = 30	Combination of restoration-oriented and loss-oriented group *N* = 28	Control group *N* = 30
Age M(SD)	36.1(9.93)	35(10.14)	39.6(11.10)	34.9(9.39)	34.7(8.52)
Women N(%)	94 (81)	24(85.7)	24(80)	22(78.6)	24(80)
Years of refugee status M(SD)	11.34 (9.95)	9.7(4.34)	10.4(3.44)	13.8(19.4)	11.3(1.27)
Relation to deceased N(%)					
Family		25(89.3)	30(100)	27(96.4)	28(93.3)
Close friend		3(10.7)	0(0)	1(3.6)	2(6.7)
Cause of death N(%)					
Violence and conflict-related		6(21.4)	7(23.3)	6(21.4)	7(23.3)
Medical condition		20(71.4)	16(53.3)	15(53.6)	18(60)
Accidents		1(3.6)	6(20)	6(21.4)	3(10)
Unknown		1(3.6)	1(3.3)	1(3.6)	2(6.7)

The main inclusion criteria to the present study were an overall score for prolonged grief disorder above the cut-off point of the Traumatic Grief Inventory-Self Report Plus (TGI-SR+), Arabic proficiency, refugee status in a transition stage of asylum-seeking, and a minimum of one year since the death of a close person.

### Recruitment and Procedure

Data were collected from May to September 2024. Participants were recruited through advertisements in organizations offering services for refugees. Participants willing to participate accessed the participation link through a QR code or the contact information provided in the advertisement. They received an identification code to ensure anonymization of the data collection.

Following the screening phase, participants who met the inclusion criteria provided online informed consent and responded to the baseline online survey (T1). Randomization allocation (1:1 ratio) was based on two equal stratified blocks of age and gender using a sequentially numbered randomization list through the Sealed Envelope online platform (Sealed Envelope Ltd, 2022). Participants who were assigned to the experimental groups received one live online psychoeducation session by trained psychologist about EW and task’s structure. Subsequently, daily tasks were sent via e-mail or SMS to each participant across one week based on the related group conditions. Upon task completion, all participants completed a post-intervention online survey (T2) and a one-week follow-up online survey (T3). They received the online links to the surveys by the principal investigator via e-mail. The primary outcome variables were PGD, PMH, depression, and anxiety.

Participants were assigned to one of four groups: the first experimental group, the writing task focused on loss-oriented stressors, the second on restoration-oriented stressors, and the third on a combination of both. The control group did not receive any specific tasks.

**Fig. 2:**
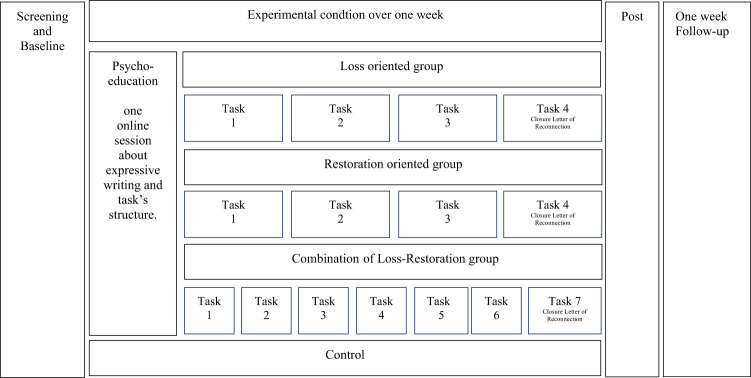
This diagram shows the study flow, including one psychoeducation session and daily expressive writing tasks lasting 15–20 min for the experimental groups and no tasks for the control group. Assessment time points include a baseline screening and pre-assessment, post-assessment, and a one-week follow-up

The daily task, length of 15–20 min, was completed over one week in a home-based environment. A guided sheet was sent sequentially via SMS or e-mail. As a final task, participants in the experimental groups wrote a “Closure Letter of Reconnection” to the deceased. For more details, see Fig. 2, study Diagram and Annex 1, Experimental Conditions.

## Measures

### Traumatic Grief Inventory-Self Report Plus (TI-SR+)

To measure PGD symptoms, the Traumatic Grief Inventory-Self Report Plus (TGI-SR+)(Lenferink et al. 2022) was used. A score above the 71 overall cut off point indicates disturbed prolonged grief disorder (Lenferink et al. 2022). The scale comprises 22 unidimensional items (e.g., “I had a desire to die in order to be with the deceased ”) which are rated on a five-point Likert-type scale (1 = never, 5 = always). Higher scores indicate higher levels of PGD. Current scale reliability was Cronbach’s α = 0.85 at T1, α = 0.92 at T2, and α = 0.93 at T3.

### Positive Mental Health Scale (PMH-Scale)

PMH was measured using the Positive Mental Health Scale (PMH-Scale) designed to assess well-being (Almubaddel 2022; Lukat et al. 2016; Velten et al. 2022). I It is a unidimensional self-report 9-item instrument with a 4-point Likert-type scale (0 = disagree, 3 = agree). An example item is"I enjoy my life “. Current scale reliability was α = 0.66 at T1, α = 0.76 at T2, and α = 0.76 at T3.

### Depression Anxiety Stress Scales 21 (DASS-21)

Depression and anxiety symptoms were assessed using the Depression Anxiety Stress Scales 21 (DASS-21) (Lovibond and Lovibond 1995; Mohammad 2021; Moussa et al. 2017). It is a 21-item scale with 7-items for each subscales. The overall score is rated based on a 4-point Likert-type scale (0 = did not apply to me at all, 3 = applied to me very much or most of the time). In the current study, the depression and anxiety subscales were used. An example item of the depression subscale is “I felt that life was meaningless”, and of the anxiety subscale is “I felt scared without any good reason”. The current scale reliability was α_depression_ = 0.86 and α_anxiety_ = 0.83 at T1, α_depression_ = 0.92 and α_anxiety_ = 0.93 at T2, and α_depression_ = 0.92 and α_anxiety_ = 0.93 at T3.

### Statistical Analysis

Statistical analyses were conducted using version 28 of the Statistical Software Package for Social Sciences (SPSS). A repeated analysis of variance (ANOVA) was performed to identify main effects (group and time) and interaction effects (group x time). A mixed design was applied: between-subjects and within-subjects, and between three assessment time points. Partial eta-squared (η2p) served as effect-size measure. Values ≥ 0.01 were considered as small, values ≥ 0.06 as medium, and values ≥ 0.14 as large effects (Cohen 1988).

The Greenhouse-Geisser correction (ε) was applied to address the violation of the sphericity assumption and the post-hoc multiple-group comparisons were Bonferroni-corrected.

## Results

Table 2 shows descriptive statistics of the outcome variables across the three measurement time points for each study group.

**Table 2:** Outcome variables descriptive statistics across time points

		Baseline		Post-intervention		Follow-up	
Variable	Group conditions	*M(SD)*	α	*M(SD)*	α	*M(SD)*	α
PGD	Total	4.03(0.518)	0.857	3.72(0.706)	0.929	3.67(0.723)	0.936
	Loss oriented	3.96(0.502)	0.830	3.73(0.576)	0.873	3.69(0.580)	0.886
	Restoration oriented	3.87(0.444)	0.793	3.49(0.621)	0.911	3.53(0.818)	0.955
	Combination of Loss-Restoration	4.09(0.532)	0.878	3.65(0.758)	0.942	3.58(0.754)	0.945
	Control	4.20(0.550)	0.887	3.98(0.787)	0.950	3.85(0.724)	0.937
PMH	Total	2.23(0.500)	0.665	2.36(0.520)	0.766	2.37(0.515)	0.766
	Loss oriented	2.27(0.553)	0.726	2.46(0.418)	0.584	2.57(0.368)	0.506
	Restoration oriented	2.34(0.407)	0.462	2.53(0.309)	0.320	2.40(0.433)	0.650
	Combination of Loss-Restoration	2.25(0.465)	0.628	2.34(0.492)	0.766	2.42(0.517)	0.789
	Control	2.09(0.546)	0.715	2.10(0.689)	0.883	2.11(0.617)	0.845
Depression	Total	2.98(0.623)	0.859	2.66(0.768)	0.919	2.61(0.730)	0.915
	Loss oriented	2.99(0.599)	0.841	2.67(0.749)	910	2.66(0.616)	0.872
	Restoration oriented	2.80(0.644)	0.874	2.43(0.624)	0.835	2.51(0.712)	0.901
	Combination of Loss-Restoration	3.06(0.568)	0.828	2.49(0.866)	0.967	2.42(0.798)	0.945
	Control	3.06(0.677)	0.873	3.00(0.721)	0.917	2.85(0.750)	0.916
Anxiety	Total	2.85(0.629)	0.833	2.63(0.830)	0.927	2.63(0.809)	0.928
	Loss oriented	2.75(0.630)	0.791	2.70(0.851)	0.932	2.70(0.717)	0.878
	Restoration oriented	2.63(0.716)	0.890	2.38(0.843)	0.913	2.48(0.941)	0.947
	Combination of Loss-Restoration	2.97(0.498)	0.745	2.49(0.821)	0.938	2.52(0.804)	0.944
	Control	3.04(0.597)	0.833	2.91(0.745)	0.914	2.81(0.763)	0.929

M = mean; SD = standard deviation

The ANOVAs were conducted to identify significant differences within the groups between the repeated measurements. Results showed a statistically significant within-subject effect over three time points on PGD, *F*(1.66, 186.66) = 22.22, *p* <.001, with a large effect size η2_p_ = 0.166, and on PMH, *F*(1.83, 205.83) = 4.61, *p* =.013, with a small effect size η2_p_ = 0.04. A significant effect was also observed on depression, *F*(1.67,187.72) = 20.29, *p* <.001, with a large effect size η2_p_ = 0.15, and on anxiety, *F*(1.79,201.41) = 8.90, *p* <.001, with a medium effect size η2_p_ = 0.07.

Considering the significant results, post hoc, Bonferroni-adjusted pairwise comparisons were conducted, including the three time points, with effect sizes reported as Cohen’s *d*. Results showed that the combination of loss and the restoration-oriented group has significant effects over the three time points on PGD between T1 and T2 (*p =*.005, *d* = 0.44) and between T1 and T3 (*p* <.001, *d* = 0.50). Moreover, a significant result was observed on depression between T1 and T2 (*p* <.001, *d* = 0.56), and T1 and T3 (*p* <.001, *d* = 0.63), and anxiety between T1 and T2 (*p =*.003, *d* = 0.47), and between T1 and T3 (*p =*.002, *d* = 0.45). In addition, the restoration-oriented group showed a significant effect only on PGD; between T1 and T2 (*p =*.018, *d* = 0.38), and between T1 and T3 (*p =*.036, *d* = 0.33).

The differences between groups analysis were significant only for PMH, *F*(3, 112) = 4.646, *p* =.004, η2_p_ = 0.111. It was not significant for PGD, *F*(3, 112) = 2.589, *p* =.056, for depression, *F*(3, 112) = 2.552, *p* =.059, and for anxiety, *F*(3, 112) = 2.128, *p* =.101. Further the investigation of between groups pairwise comparison for PMH revealed significant differences between restoration-oriented and control group, with higher levels of PMH in the restoration-oriented group at T2 (*d*=−0.432, *p* =.008). Furthermore, the loss-oriented group revealed maintained higher levels of PMH at T2 (*d*=−0.363, *p* =.035) and T3 (*d*=−0.452, *p* =.003) compared to the control group.

**Fig. 3:**
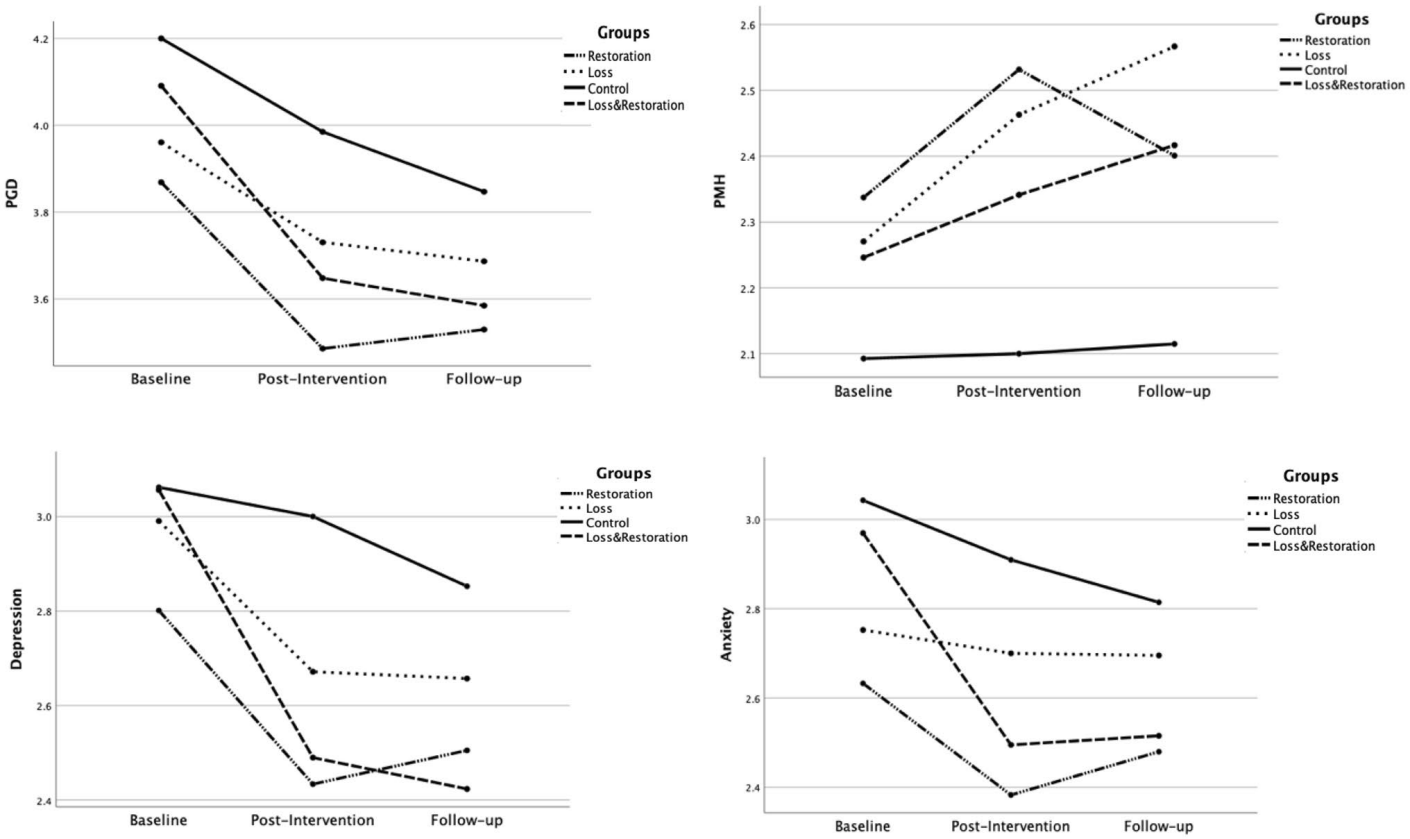
This figure presents the results of the repeated measures ANOVA, showing the interaction effects of time (baseline, post-intervention, and one-week follow-up) and group (Restoration-Oriented Group, Loss-Restoration Oriented Group, and Control Group) on Prolonged Grief Disorder (PGD), Positive Mental Health (PMH), depression, and anxiety; Include the N of the groups

The results considering the interaction effect between the three time points and different group conditions shown in Fig. 3 were not statistically significant for PGD, *F*(5.000, 186.668) = 0.646, *p* =.665, PMH *F*(5.513, 205.830) = 1.154, *p* =.333, depression, *F*(5.028, 187.725) = 1.761, *p* =.122, and anxiety, *F*(5.395, 201.411) = 1.490, *p* =.190.

## Discussion

The current study investigated the effects of guided EW on PGD, depression, anxiety, and PMH in bereaved refugees in the transition stage. Our findings showed a significant between-group effect on PMH at post-intervention and one-week follow-up and a significant within-subject effect on PGD, depression, anxiety, and PMH. However, the interaction results between groups per time were not significant. Results indicate that the inconsistent improvement patterns between the experimental groups and within subjects over time had an immediate but not durable effect.

The restoration-oriented group showed a significant but not durable effect compared to the other groups. It is important to note that based on DPM, the restoration term is about secondary stressors related to life after the loss (Stroebe and Schut 1999). The findings indicate that focusing only on the secondary stressors of adaptive requirements of daily life after loss can improve PMH and reduce PGD. However, the improvement is not persistent. This is consistent with previous research describing that avoidance of the primary issues related to the death event can influence the outcome of grief treatment (Boelen 2021). It also aligns with the DPM coping with bereavement, in which the dynamic of coping implies confronting both primary stressors related to the loss event and secondary stressors related to life after loss (Stroebe and Schut 1999, 2010).

In contrast, the group that applied the loss-oriented tasks, focusing on the primary stressors related to the death event, showed more durable significant effect only on PMH, as a reflection of psychological well-being and an important outcome to assess the effectiveness of interventional studies (Brailovskaia et al. 2020; Margraf et al. 2024). These findings regarding the role of EW within the refugee grief context confirm the previously reported effectiveness of EW as a stand-alone intervention for decreasing distress and improving psychological well-being (Den Elzen et al. 2023; Ruini and Mortara 2022). However, the loss-oriented task had no effect on PGD, depression, and anxiety, which might need more specialized intervention. This is also confirmed in previous research that showed a positive but not durable effect of brief interventions on refugee well-being (Daniel et al. 2024).

The improvement in PGD is linked to the context of death, which can influence the effectiveness of interventions (Hilberdink et al., 2023). Refugees experience ongoing challenges (Miller and Rasmussen 2017), and stress related to their asylum-seeking state (Miller and Rasmussen 2017; Taylor et al. 2024; Vukčević Marković et al. 2021), which can affect their well-being and prolong their grieving state. In transition contexts, individuals prioritize daily basic needs (Garsow et al. 2021; Rasmussen & Annan, 2009) this potentially can lead to death event-related grief confrontation avoidance (Stroebe and Schut 1999) and increase the risk of prolonged grief. Addressing the oscillation between post-death daily life challenges and the loss itself is essential in designing effective interventions for bereaved refugees. Applying the combination of loss and restoration-oriented tasks leads to more persistent improvement in PGD, depression, and anxiety at post and follow-up.

Despite the previously mentioned significant effect, the results showed a non-significant interaction between time and different group conditions, which indicates inconsistent improvement patterns between the experimental groups and within subjects over time.

The inconsistent patterns can be attributed to the retrospective fluctuation characteristic of PGD symptoms over time (Lenferink et al. 2024). The nature of the grief experience might produce similar patterns of PGD symptoms between groups over short follow-up assessments with a probable retrospective and not steady direction of the fluctuation (Arizmendi & O’Connor, 2015; Lenferink et al. 2024). Furthermore, the nonpersistent effect over time could be linked to the short intervention duration. More extended application of EW tasks could provide more accurate results on the efficacy of EW. This is also consistent with previous research about the effect of EW durability, which confirms its short-term improvements in clinical symptoms (Sloan et al. 2009).

The current study has several limitations linked to its findings. First, the guided self-help design should be integrated with specialized mental health services when accessible. Second, the self-reporting and quantitative assessment can be improved by including additional qualitative data that address the content of the writing tasks. Third, the short intervention and follow-up periods limit the generalizability of the results. Fourth, addressing refugees only in the transition stage can be extended to other stages.

The results provide a scalable intervention for bereaved refugees in a transition stage. Guided self-help tools can improve accessibility to mental health services, overcome language and cultural barriers, and prevent mental health deterioration. The availability of such immediate interventions during early grieving experiences can foster healthy grief work. Future research can compare this intervention with other self-help interventional approaches as well as prolonged intervention duration and follow-up.

## Supplementary Information

Below is the link to the electronic supplementary material.

Supplementary Material 1

## Data Availability

The dataset and further material analysed during the current study will be available from the corresponding author upon reasonable request.The study has a preregistration within the Open Science Framework (https://osf.io/vxz5d/?view_only=b891e2bae2164d3ea558134c2f1c627b).
