# Supplementary material for: The Effectiveness of Online Thematic Expressive Writing on Prolonged Grief Disorder, Anxiety, Depression, and Positive Mental Health among Refugees in the Transition Stage of Asylum Seeking
Source: J Immigr Minor Health. 2025 Sep 12;28(2):360–8. doi: 10.1007/s10903-025-01778-8 (PMC13083514; doi:10.1007/s10903-025-01778-8)
Supplement: Supplementary file 1 — Supplementary Material 1 [file 10903_2025_1778_MOESM1_ESM.docx]

**Annex 1**

**Experimental Conditions**

The group conditions were designed based on an EW intervention approach using the Pennebaker paradigm (Pennebaker, 2004, 2010; Ruini & Mortara, 2022). This approach can be applied as a standalone treatment approach that facilitates the expression of emotions and thoughts through written words. In the current study, to facilitate the expression of distress related to bereavement, guiding questions for the daily writing tasks were combined with content from the DPM of coping with bereavement (Stroebe & Schut, 1999). Participants were assigned to one of four groups: the first experimental group, the writing task focused on loss-oriented stressors, the second on restoration-oriented stressors, and the third on a combination of both. The control group did not receive any specific tasks.

The daily task, length of 15-20 minutes, was completed over one week in a home-based environment. A guided sheet was sent sequentially SMS or e-mail. As a final task, participants in the experimental groups wrote a "Closure Letter of Reconnection" to the deceased. The written content was not collected and remained for each participant's private use. For more details, see Figure 2, study Diagram.

Given the ethical considerations related to the vulnerable population, referrals for psychological first aid were available upon request. During the initial psychoeducation session, participants were informed about this procedure and when it is recommended; the control group was informed through the information sheet.

**The Intervention**

The writing intervention was divided into three phases. The initial phase is a psychoeducation session, followed by the implementation of the writing tasks and a final task of closure letter.

**The Psychoeducation Session**

A one-hour online session was conducted by a trained psychologist. The session introduced the concept of expressive writing, the structure of the writing tasks, and information regarding the availability of psychological first aid and referrals, including when it is recommended to seek additional support.

**The Writing Tasks**

The writing tasks were conducted in a home-based context. Each participant received a daily guidance instruction sheet in consecutive day’s task, sent via SMS or email according to their preference. The tasks required 15–20 minutes to complete each day and focused on a different theme based on the randomized group assignment over the course of one week. Participants were free to choose the timing and setting for completing the task. The written content was not shared with the research team.

The structure of the task sheets followed the Pennebaker paradigm, with adaptations to the instructions and orientation based on the daily content focus.

The content of the tasks was linked to the dual model of coping with bereavement, including three categories: Loss-oriented coping addresses the confronting process with coping aspects of the loss itself, such as yearning, sadness, and issues related to the relationship with the deceased and the pain of loss., restoration-oriented coping is associated with life changes and challenges as a consequence of the death event, such as assuming new roles among the family and outside, new life skills requirements, and new relationships, and the third category of tasks involving a combination of both.

**The closure letter**

The final task for all groups included a closure letter addressed to the deceased to help achieve a coherent narrative and closure around exposure experiences through the writing tasks.
